# Contrast-enhanced Ultrasound Features of Intrahepatic Cholangiocarcinoma: A New Perspective

**DOI:** 10.1038/s41598-019-55857-6

**Published:** 2019-12-18

**Authors:** Tianjiao Chen, Xiaoyan Chang, Ke Lv, Yong Wang, Xianshui Fu, Li Tan, Yang Gui, Tongtong Zhou, Xueqi Chen, Yuxin Jiang

**Affiliations:** 1 Department of Ultrasound, Peking Union Medical College Hospital, Peking Union Medical College, Chinese Academy of Medical Sciences, Beijing, 100730 China; 2 Department of Pathology, Peking Union Medical College Hospital, Peking Union Medical College, Chinese Academy of Medical Sciences, Beijing, 100730 China; 3 Department of Diagnostic Ultrasound, Cancer Hospital, Chinese Academy of Medical Sciences, Peking Union Medical College, Beijing, 100021 China; 4 Department of Ultrasound, First Affiliated Hospital of PLA General Hospital, Beijing, 100048 China

**Keywords:** Liver cancer, Liver cancer

## Abstract

The objective of this study was to illustrate our specific findings for intrahepatic cholangiocarcinoma (ICC) lesions on contrast-enhanced ultrasound (CEUS). In this study, 21 patients at our hospitals with pathologically proven ICC and CEUS data were retrospectively enrolled. General clinical data of the patients, and features of lesions on conventional and contrast-enhanced ultrasound were recorded. Two experienced radiologists retrospectively reviewed all images by consensus. On gray-scale sonography, hypoechoic, isoechoic and hyperechoic lesions accounted for 85.7%, 9.5% and 4.8%, respectively, of all lesions. Hypovascular patterns were found for 95.2% of the lesions on color Doppler flow imaging. During the arterial phase of CEUS, heterogeneous hyperenhancement, homogeneous hyperenhancement, rim-like hyperenhancement, isoenhancement and hypoenhancement were observed for 61.9%, 19.0%, 9.5%, 4.8%, 4.8% of the lesions, respectively. During the portal venous and late phases, 85.7% and 95.2% of the lesions, respectively, exhibited hypoenhancement. In addition, 66.7% of the ICC lesions exhibited washed-out interiors but little decrease in enhancement at the periphery during the portal venous phase, resulting in the formation of a hyperenhanced peripheral rim. In conclusion, the rim sign in the portal venous phase of CEUS could help diagnose ICC. This trait could be related to the infiltrating growth pattern of ICC.

## Introduction

Intrahepatic cholangiocarcinoma (ICC) is a relatively rare type of primary liver cancer that arises from the intrahepatic bile duct epithelium^1,2^. However, the incidence and mortality of ICC are believed to have progressively increased over the past several decades^3–7^. The diagnosis of ICC plays an increasingly important role but remains highly challenging. In previous studies, certain researchers have suggested that peripheral rim-like enhancement during the arterial phase of contrast-enhanced ultrasound (CEUS) occurred in the majority of ICC lesions^8–12^. Whereas other results have indicated that this feature could be overestimated or based on a certain condition^13–17^. The issue of whether ICC has specific characteristics during CEUS examination remains controversial. In this study, we describe our specific findings for ICC on CEUS.

## Methods

### Patients

Between December 2004 and August 2019, 21 patients at our hospitals with pathologically proven ICC (verified via surgical specimen for 18 patients and via biopsy for 3 patients) and CEUS data were retrospectively enrolled in this study. Exclusion criteria included the presence of a combination of hepatocellular carcinoma and cholangiocarcinoma; uncertainty regarding whether lesions observed via CEUS matched the pathologically verified lesions; and the treatment of lesions prior to CEUS. Patients’ ages, symptoms and laboratory test results were recorded before therapy. Follow-up periods ranged from 0.5 to 87 months. The Institutional Review Board of Peking Union Medical College Hospital approved this retrospective study and waived the requirement for written informed consent.

### Contrast agent

SonoVue^®^ (Bracco, Italy), which is sulfur hexafluoride with a phospholipid shell, was used as the contrast agent. During CEUS examination, an intravenous bolus injection of SonoVue^®^ at a dose of 2.4 ml was first administered, followed by a flush with 5 ml of normal saline.

### Conventional ultrasound and CEUS examinations

Conventional ultrasound examination was performed first. Features of patients’ lesions, including location, number, size, echogenicity and vascularization, were observed and recorded during gray-scale and color Doppler sonography. After the target lesion had been confirmed, CEUS examination was performed using a low mechanical index (<0.1) technique. A timer was started when the contrast agent was injected. CEUS images were recorded for more than four minutes after contrast agent injection.

### Image analysis

During CEUS examinations, the arterial, portal venous and late phases were defined as <30 s, 31–120 s and >120 s after contrast agent injection, respectively. Enhancement was classified as hyperenhancement, isoenhancement, or hypoenhancement relative to adjacent liver parenchyma. In accordance with various enhancement manifestations that were observed, enhancement patterns in the arterial phase were further categorized as homogeneous enhancement (uniform enhancement of the whole lesion), heterogeneous enhancement (different degrees of enhancement throughout the lesion), and peripheral rim-like enhancement (enhancement that was primarily limited to the periphery of the lesion). Two experienced radiologists retrospectively reviewed all images by consensus. Software QontraXt v.3.06 (Esaote, Italy) was used for quantitative analysis. One region of interest (ROI) was selected in the interior of the lesion, which showed rapid hyperenhancement during the arterial phase and decrease in enhancement during the portal venous phase. Another region of interest was selected at the periphery of the lesion which showed rapid hyperenhancement during the arterial phase and decrease in enhancement during the late phase. Then the parametric curves of the two ROIs were compared.

## Results

### Patient characteristics

There were 14 male patients and 7 female patients (mean age 58.3 ± 12.2 years; age range 33–83 years). ICC was discovered incidentally during physical examination in 12 patients. With respect to manifestations, 6 patients complained of abdominal discomfort; other symptoms included back pain, nausea and weakness. Positivity for hepatitis B surface antigen (HBsAg) was detected in 5 patients. 4 patients had elevated carbohydrate antigen (CA) 19-9 levels (41–17871 U/ml; 0–37 U/ml). Elevated alpha-fetoprotein (AFP) (112.6 ng/ml; 0–20 ng/ml) and carcinoembryonic antigen (CEA) (18.3 ng/ml; 0–5 ng/ml) levels were detected in only one patient respectively. Overall, 27.8% (5/18) of the patients experienced recurrence after surgical resection, and 33.3% (7/21) of the patients had died.

### Imaging findings

Only one patient had two ICC lesions; the remaining patients had just one lesion. For the patient with two lesions, only the larger lesion was analyzed. Only 1 lesion was in the caudate lobe of the liver, whereas 8 lesions were in the right lobe, and 11 lesions were in the left lobe. There was another lesion located in the right lobe, left lobe as well as the caudate lobe of the liver. Among the 19 lesions that were histologically classified, 1 lesion was well differentiated; 1 lesion was well to moderately differentiated; 6 lesions were moderately differentiated; 5 lesions were moderately to poorly differentiated; and 6 lesions were poorly differentiated. The largest diameters of the ICC lesions ranged from 2.8 cm to 13.7 cm (mean, 5.3 ± 2.4 cm). On conventional ultrasound, 85.7% (18/21), 9.5% (2/21) and 4.8% (1/21) of the lesions were hypoechoic, isoechoic and hyperechoic, respectively. Most of the lesions (95.2%, 20/21) exhibited sparse or little color Doppler signal (Table 1). Intrahepatic cholangiectasis was detected in 3 patients.

**Table 1:** Conventional US Characteristics of 21 ICC Lesions.

	Echogenicity		
	Hypoechoic (n = 18)	Isoechoic (n = 2)	Hyperechoic (n = 1)
Mean nodule size (cm)	5.4 ± 2.6	4.4 ± 0.7	4.7
Blood supply (n)			
relatively abundant	1	0	0
sparse or little	17	2	1

**Figure 1:**
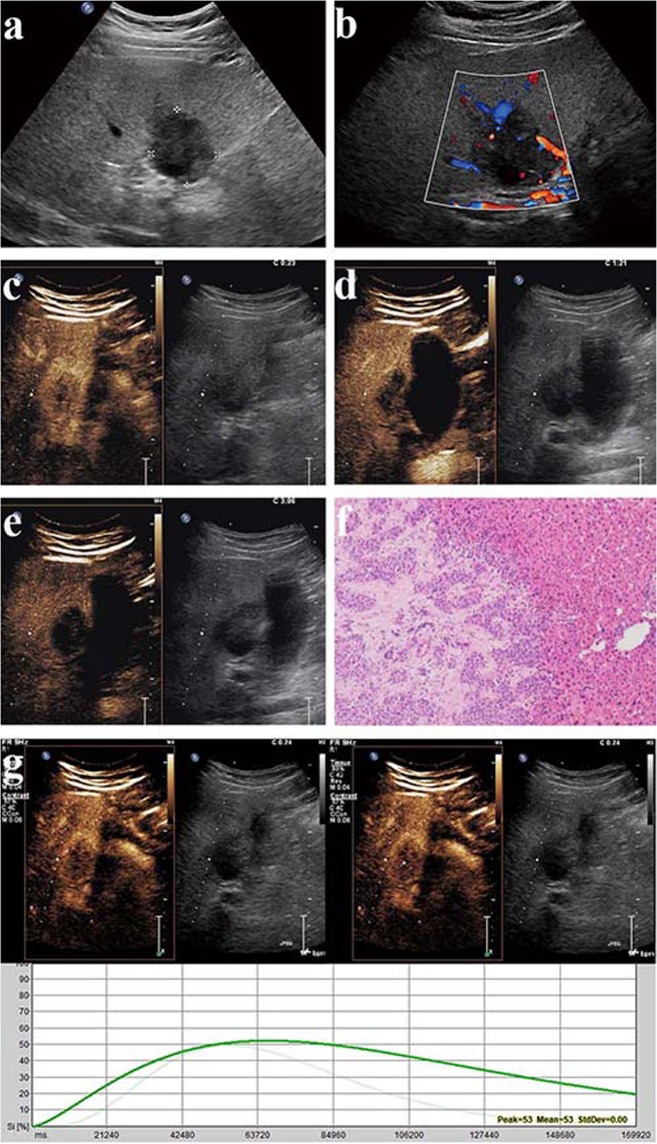
Intrahepatic cholangiocarcinoma (ICC) in a 62–year-old male. (see Supplementary Video S1). (**a**) Conventional ultrasound shows a hypoechoic lesion with a size of 4.2 cm × 3.5 cm in the left lobe of the liver (cursors); (**b**) Color Doppler sonography indicates a relatively sparse blood supply; (**c**) During the arterial phase (23 s), the lesion exhibits heterogeneous hyperenhancement; (**d**) During the portal venous phase (81 s), the lesion exhibits hypoenhancement of its interior with hyperenhancement in the peripheral region, resulting in the formation of a hyperenhanced rim; (**e**) During the late phase (186 s), the lesion exhibits hypoenhancement; (**f**) Micrograph (original magnification, ×100; hematoxylin and eosin stain) reveals that the periphery of the tumor is irregular and intermingled with the adjacent normal hepatic tissue. The lesion shows an infiltrative characteristic. (**g**) The parametric curves reveal perfusion of the region of interest in the interior (light green, Peak = 50.2, Sharpness = 0.074 1/s) and periphery (dark green, Peak = 53.1, Sharpness = 0.024 1/s) of the lesion from 10 s to 180 s.

**Figure 2:**
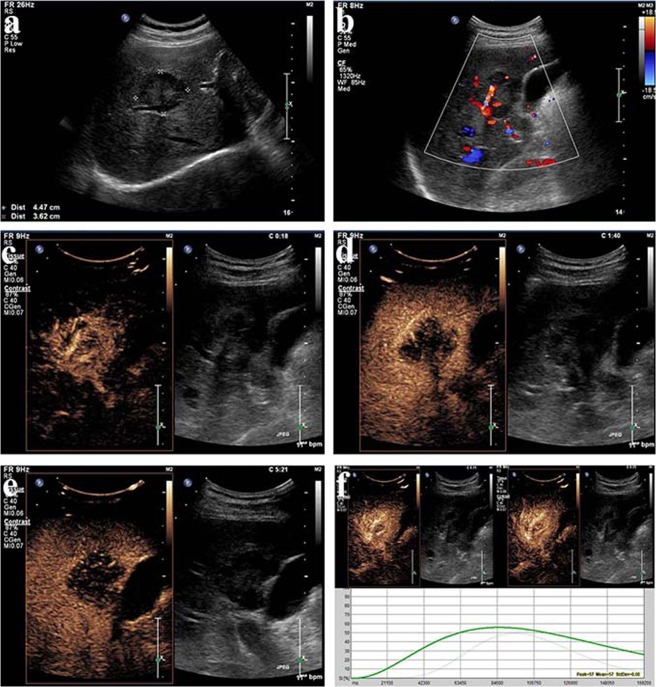
Intrahepatic cholangiocarcinoma (ICC) in a 54–year-old male. (see Supplementary Video S2). (**a**) Conventional ultrasound shows a hypoechoic lesion with a size of 4.5 cm × 3.6 cm near the hepatic hilar region (cursors); (**b**) Color Doppler sonography indicates a relatively abundant blood supply; (**c**) During the arterial phase (18 s), the lesion exhibits heterogeneous hyperenhancement; (**d**) During the portal venous phase (100 s), the lesion exhibits hypoenhancement of its interior with hyperenhancement in the peripheral region, resulting in the formation of a hyperenhanced rim; (**e**) During the late phase (321 s), the lesion exhibits hypoenhancement; (**f**) The parametric curves reveal perfusion of the region of interest in the interior (light green, Peak = 50.0, Sharpness = 0.105 1/s) and periphery (dark green, Peak = 56.8, Sharpness = 0.030 1/s) of the lesion from 10 s to 180 s.

**Figure 3:**
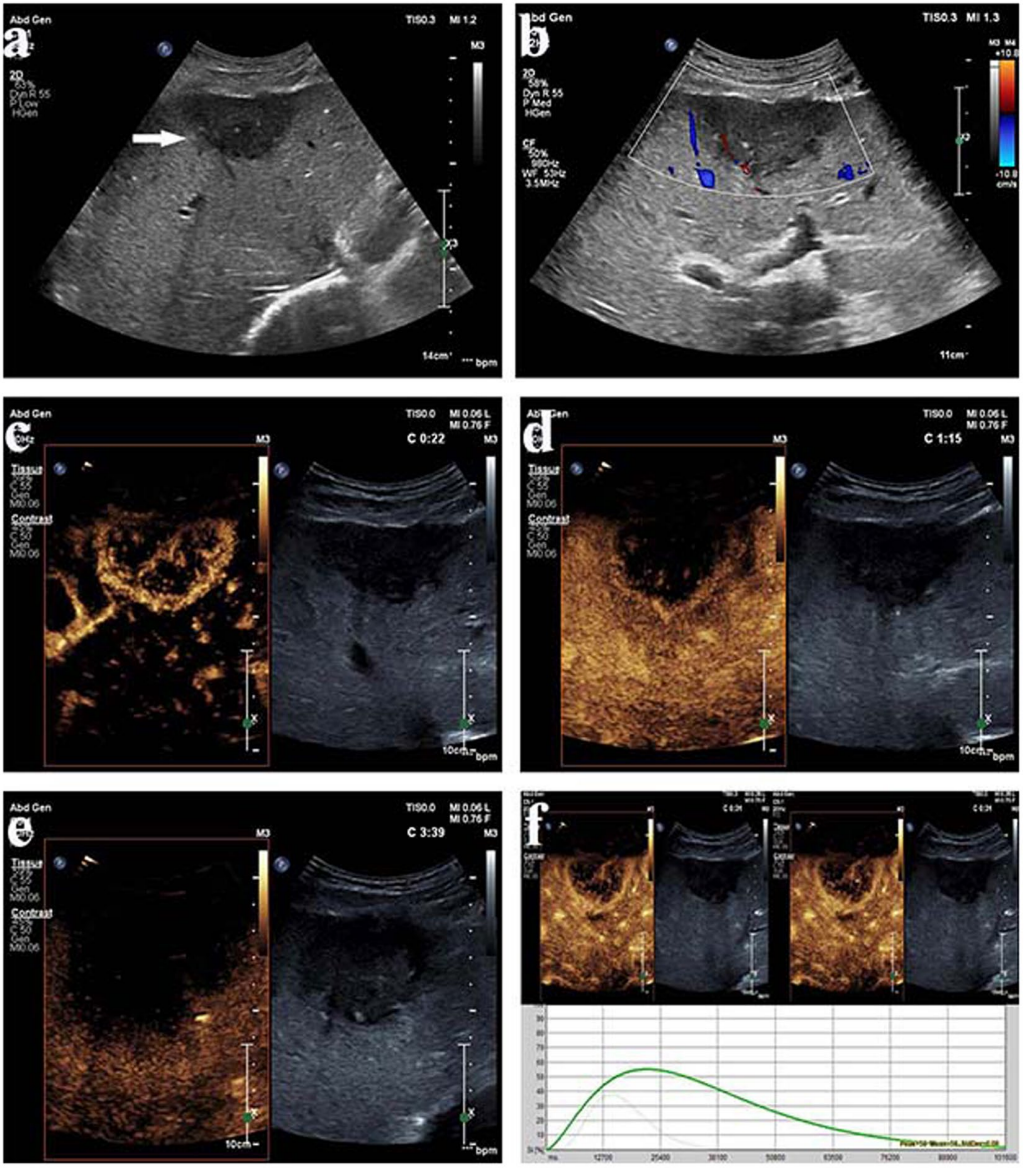
Intrahepatic cholangiocarcinoma (ICC) in a 83–year-old male. (see Supplementary Video S3–4). (**a**) Conventional ultrasound shows a hypoechoic lesion with a size of 5.5 cm × 3.1 cm in the left lobe of the liver (arrow); (**b**) Color Doppler sonography indicates a relatively sparse blood supply; (**c**) During the arterial phase (22 s), the lesion exhibits peripheral rim-like enhancement; (**d**) During the portal venous phase (75 s), the lesion exhibits hypoenhancement of its interior with hyperenhancement in the peripheral region, resulting in the formation of a hyperenhanced rim; (**e**) During the late phase (219 s), the lesion and its surrounding area exhibit hypoenhancement; (**f**) The parametric curves reveal perfusion of the region of interest in the interior (light green, Peak = 38.5, Sharpness = 0.391 1/s) and periphery (dark green, Peak = 56.0, Sharpness = 0.075 1/s) of the lesion from 10 s to 112 s.

On CEUS, the most common enhancement pattern during the arterial phase was heterogeneous hyperenhancement, which was observed for 61.9% (13/21) of the lesions, followed by homogeneous hyperenhancement (19.0%, 4/21), rim-like hyperenhancement (9.5%, 2/21), isoenhancement (4.8%, 1/21) and hypoenhancement (4.8%, 1/21). During the portal venous phase, 85.7% (18/21) of the lesions exhibited hypoenhancement, and the remaining lesions exhibited isoenhancement. During the late phase, only 4.8% (1/21) of the lesions remained isoenhanced, whereas the remaining lesions were hypoenhanced (Table 2). All lesions showed decrease in enhancement during the portal venous and late phases, albeit to varying degrees. We also found a similar pattern for many lesions during the portal venous phase. In particular, these lesions exhibited washed-out interiors but little decrease in enhancement at the periphery. In other words, these lesions presented with hypoenhanced interiors but a hyperenhanced peripheral rim during the portal venous phase. During the late phase, this peripheral hyperenhancement gradually decreased until the entire lesion exhibited hypoenhancement. The aforementioned manifestations were observed for 66.7% (14/21) of the examined lesions (Figs. 1–3, see Supplementary Video S1–4).

**Table 2:** CEUS Manifestations of 21 ICC Lesions.

	Arterial phase	Portal venous phase		Late phase
		With rim sign	Without rim sign	
Heterogeneous hyperenhancement (n)	13	0	0	0
Homogeneous hyperenhancement (n)	4	0	0	0
Rim-like hyperenhancement (n)	2	0	0	0
Isoenhancement (n)	1	1	2	1
Hypoenhancement (n)	1	13	5	20

## Discussion

**Figure 4:**
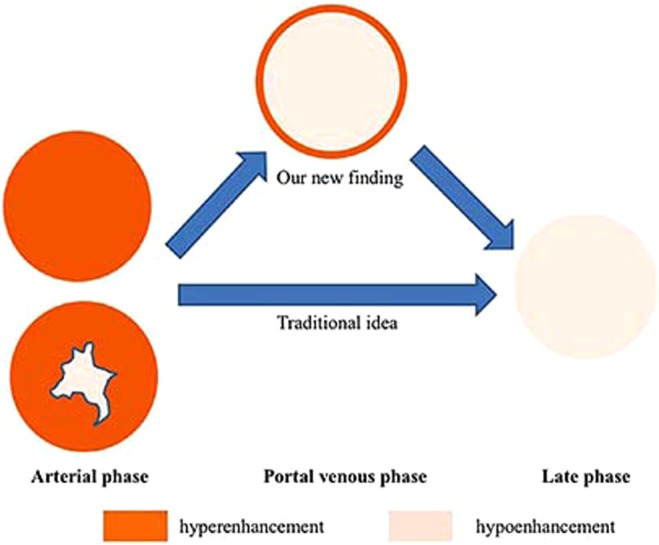
A sketch of the rim sign in the portal venous phase during CEUS examination. 66.7% of the ICC lesions in our study exhibited a lasting hyperenhanced peripheral rim during the portal venous phase of CEUS. These lesions exhibited washed-out interiors but little decrease in enhancement at the periphery during the portal venous phase, no matter what the enhancement pattern was during the arterial phase. Then a gradual decrease in enhancement occurred during the late phase.

In our study, 66.7% of the ICC lesions exhibited a lasting hyperenhanced peripheral rim during the portal venous phase of CEUS. We called this character as the rim sign in the portal venous phase. These lesions exhibited washed-out interiors but little decrease in enhancement at the periphery during the portal venous phase, no matter what the enhancement pattern was during the arterial phase. Then a gradual decrease in enhancement occurred during the late phase (Fig. 4). This characteristic was different from the peripheral rim-like enhancement during the arterial phase mentioned in previous studies. To our knowledge, it had not been presented before and we thought that the features described above could aid in the differential diagnosis of ICC from hepatocellular carcinoma (HCC).

Cholangiocarcinoma is the second most common primary hepatic cancer and accounts for 10–20% of all primary hepatic tumors^4,18^. ICC has been described as cholangiocarcinoma located proximally to the second-degree bile ducts^19^. ICC accounts for less than 10% of cholangiocarcinoma cases^18,19^. A meta-analysis has indicated that cirrhosis, chronic hepatitis B, chronic hepatitis C, alcohol use, obesity, and diabetes are major risk factors for ICC^20^. Other suspected risk factors include biliary malformations such as primary sclerosing cholangitis, cholelithiasis, choledocholithiasis, hepatolithiasis, inflammatory bowel disease, choledochal cysts, smoking, and genetic polymorphisms^4,7,16^.

ICC is typically asymptomatic and discovered incidentally via imaging examinations^6,7^. ICC patients may also complain of abdominal pain or nonspecific symptoms such as fatigue, weakness, and weight loss^6,7^. Several tumor markers may be elevated, but such markers do not exhibit high sensitivity or specificity for ICC^1,6^. Elevated AFP levels are observed in only a small percentage of ICC patients^21^. The sensitivities of CA19-9 and CEA for ICC are 50–63% and 15–68%, respectively^6^. However, enhanced sensitivity and specificity can be achieved by utilizing a combination of both markers^5^. Although various treatment options exist for ICC, complete surgical resection is traditionally the ideal therapeutic approach^1,5^. However, few patients are candidates for this treatment^1,6^. The prognoses of ICC patients, even those who undergo complete surgical resection, are typically poor^1,5–7^. The results in our study were essentially consistent with those of prior studies.

With respect to ultrasound examination, in our study, ICC lesions were hypoechoic, isoechoic, or hyperechoic on gray-scale sonography; most of the examined lesions were hypoechoic. These findings were consistent with the results of some but not all previous investigations^8,9,13,22^. However, conventional ultrasound imaging findings for ICC are non-specific^9,10^.

On CEUS, most lesions in our study showed hyperenhancement during the arterial phase, followed by washout to hypoenhancement during the portal venous phase. This characteristic was consistent with the features of most malignant tumors of the liver^14,21^. We observed five enhancement patterns during the arterial phase, including heterogeneous hyperenhancement, homogeneous hyperenhancement, rim-like hyperenhancement, isoenhancement and hypoenhancement. In prior studies, certain authors have regarded lesions with peripheral rim-like hyperenhancement as the most common ICC lesions^8–12^, although other researchers have found that lesions with this pattern were not observed in most ICC cases or were rarely present^13–17^. In our study, most ICC lesions showed heterogeneous hyperenhancement during the arterial phase. However, it is reasonable whether heterogeneous hyperenhancement or peripheral rim-like hyperenhancement is observed for most ICC lesions, given that enhancement patterns of such lesions on CEUS may relate to tumor size and the distribution of tumor cells. Hyperenhanced areas in ICC lesions always correspond to regions with more tumor cells^8^. Small ICC lesions tend to exhibit homogeneous enhancement, whereas larger ICC lesions tend to exhibit heterogeneous or peripheral enhancement^9^. The likely cause of these findings is that tumor cells account for the majority in small ICC lesions, but that more fibrous tissues and necrosis appear as ICC lesions become larger^9,21^. Certain authors have suggested that tumor size may also influence the washout patterns that are observed. More ICC lesions demonstrated early washout (<60 s) in a study by Li *et al*. (87.9%) than in a study by Vilana *et al*. (47.6%)^16,23^. Median tumor size was larger in the former study than in the latter study (4.0 cm vs 3.2 cm)^16,23^.

Guidelines from 2012 that were produced via the cooperative efforts of the European Federation of Societies for Ultrasound in Medicine and Biology (EFSUMB), the World Federation for Ultrasound in Medicine and Biology (WFUMB) and many other institutions suggest that ICC lesions have many enhancement patterns during the arterial phase but all exhibit washout during the late phase^24^. They also indicate that typical features of cholangiocarcinoma are rim-like enhancement (central hypoenhancement) during the arterial phase, hypoenhancement during the portal venous phase and nonenhancement during the late phase^24^. In our study, all lesions showed decrease in enhancement during the portal venous and late phases, albeit to varying degrees. These findings were consistent with the common features of ICC described in the aforementioned guidelines. However, because the washout phenomenon is characteristic of malignancies, this phenomenon is not particularly specific to ICC.

In our study, 66.7% of the lesions remained hyperenhanced at the periphery during the portal venous phase, resulting in the formation of a hyperenhanced peripheral rim. Subsequently, during the late phase, lesions with this enhancement pattern exhibited hypoenhancement in their interiors and gradually decreasing enhancement of the peripheral rim. We called it as the rim sign in the portal venous phase. To our knowledge, this perspective has not previously been reported.

In our opinion, the infiltrating growth pattern associated with ICC could be related to the formation of hyperenhanced peripheral rims during the portal venous phase. The peripheral region of ICC lesions is typically the transitional area between tumor tissue and normal hepatic tissue. For such lesions, washout could be slower for peripheral regions than for the lesion interiors. As for HCCs, cirrhosis is the major clinical risk factor^2^. And the lesion often has a fibrous pseudo-capsule in the presence of cirrhosis, which leads to a well-defined border^2^. Thus, the rim sign in the portal venous phase usually does not occur in HCCs and contributes to the differential diagnosis of ICC and HCC.

The features described above could aid in the differential diagnosis of ICC, such as by helping to distinguish ICC from HCC. However, ICC lesions behave somewhat similarly to metastases on CEUS examination; therefore, metastases should first be excluded. Nonetheless, more cases need to be studied.

During CEUS examination, 66.7% of the ICC lesions in our study showed a hyperenhanced peripheral rim during the portal venous phase, followed by gradually decreasing enhancement during the late phase. If a hepatic lesion shows characteristics of malignancy and this enhancement pattern on CEUS, ICC should be taken into consideration after metastases have been clinically excluded. The observed enhancement characteristics could be related to the infiltrating growth pattern of ICC.

## Supplementary information

Supplementary Video S1

Supplementary Video S2

Supplementary Video S3

Supplementary Video S4
